# Hospitalised patients as stewards of their own antibiotic therapy: a qualitative analysis informing the strategic design of interventions to encourage shared decision-making in tertiary hospital settings in Singapore

**DOI:** 10.3389/fpubh.2024.1347764

**Published:** 2024-07-31

**Authors:** Huiling Guo, David Chien Lye, Tat Ming Ng, Jyoti Somani, Andrea Lay Hoon Kwa, Shimin Jasmine Chung, Angela Chow

**Affiliations:** ^1^Department of Preventive and Population Medicine, Office of Clinical Epidemiology, Analytics, and Knowledge, Tan Tock Seng Hospital, Singapore, Singapore; ^2^Infectious Disease Research and Training Office, National Centre for Infectious Diseases, Singapore, Singapore; ^3^Department of Infectious Diseases, Tan Tock Seng Hospital, Singapore, Singapore; ^4^Yong Loo Lin School of Medicine, National University of Singapore, Singapore, Singapore; ^5^Lee Kong Chian School of Medicine, Nanyang Technological University, Singapore, Singapore; ^6^Department of Pharmacy, Tan Tock Seng Hospital, Singapore, Singapore; ^7^Division of Infectious Diseases, National University Hospital, Singapore, Singapore; ^8^Department of Pharmacy, Singapore General Hospital, Singapore, Singapore; ^9^Programme in Emerging Infectious Diseases, Duke-NUS Medical School, Singapore, Singapore; ^10^Department of Infectious Diseases, Singapore General Hospital, Singapore, Singapore; ^11^Saw Swee Hock School of Public Health, National University of Singapore, Singapore, Singapore

**Keywords:** antimicrobial resistance, antibiotic prescribing, shared decision-making, hospitalised patients, tertiary hospital

## Abstract

**Background:**

Shared decision-making (SDM) on antibiotic therapy may improve antibiotic use in tertiary hospitals, but hospitalised patients are apprehensive about being involved in it. Understanding the facilitators and barriers to SDM can inform the design and implementation of interventions to empower these patients to engage in SDM on their antibiotic therapies.

**Methods:**

We conducted qualitative interviews with 23 adult patients purposively sampled with maximum variation from the three largest tertiary-care hospitals in Singapore (April 2019─October 2020). Thematic analysis was conducted using the Theoretical Domains Framework and Capability, Opportunity, Motivation, Behaviour (COM-B) model to identify areas for intervention.

**Results:**

Hospitalised patients lacked comprehensive knowledge of their antibiotic therapies and the majority did not have the skills to actively query their doctors about them. There was a lack of opportunities to meet and interact with doctors, and patients were less motivated to engage in SDM if they had a self-perceived paternalistic relationship with doctors, trusted their doctors to provide the best treatment, and had self-perceived poor knowledge to engage in SDM. To empower these patients, they should first be educated with antibiotic knowledge. Highlighting potential side effects of antibiotics could motivate them to ask questions about their antibiotic therapies. Environment restructuring, as facilitated by nurses and visual cues to nudge conversations, could create opportunities for interactions and motivating patients into SDM on their antibiotic therapies.

**Conclusion:**

Education and environmental restructuring should be explored to empower hospitalised patients to engage in SDM on their antibiotic therapies.

## 1 Introduction

Antimicrobial resistance (AMR) is expected to result in an annual incidence of 10 million global deaths by 2050, with nearly half of the deaths occurring in the Asian population (1). With AMR-attributable deaths already reaching 1.27 million in 2019 alone (2), there is an urgent need to ensure that antibiotics are appropriately used to mitigate this global public health threat (3).

Internationally, the use of antimicrobials in tertiary hospitals is not trivial. In a global point prevalence study conducted in 2015, 53 countries reported that 34% of patients warded in 303 study sites were receiving at least one antimicrobial (4). In Southeast Asia, in particular, more than half of the patients were receiving antibiotics during their hospital stay (5–7), with a prevalence of 51% reported in Singapore (5) – a developed country in the region.

Antibiotic stewardship programmes (ASPs) are the cornerstones of ensuring appropriate antibiotic use and reducing AMR (8–11) in tertiary hospitals through active monitoring and evaluation (12–15). However, the ASP team of a hospital often comprises a limited number of microbiologists, infectious disease doctors and pharmacists (16), who become easily overwhelmed by a large-scale epidemic or pandemic (17). New alternative strategies need to be explored to expand ASP teams beyond healthcare professionals, for example to involve hospitalised patients, to augment ongoing ASPs.

In primary care, patients play an important part in antibiotic stewardship by engaging in shared decision-making (SDM) with their doctors on antibiotic prescribing, which have been found to be effective in improving the appropriateness of antibiotics prescribed (18). In the handful of studies conducted, it was suggested that SDM on antibiotic therapy in tertiary hospitals may also yield favourable outcomes on optimising appropriate antibiotic decisions (19). However, the lack of opportunities provided by healthcare professionals to interact, as cited by hospitalised patients, have limited SDM (20, 21). In addition, the higher acuity of illness of these patients could have led to poorer confidence in them to engage in SDM with their clinical team. Furthermore, hospitalised patients have also shared doubts about their capabilities in comprehending medical information and therefore were more likely to trust their doctors to make the final antibiotic decision (21).

In order to explore the possibilities of involving hospitalised patients in future antibiotic stewardship efforts, it is imperative that we first understand the factors influencing these patients to be actively engaged in SDM for their antibiotic therapy. We aimed to use the Theoretical Domains Framework (TDF) (22) to identify the influencing factors. After which, the domains will be mapped onto the Capability, Opportunity, Motivation, Behaviour (COM-B) model (23), to identify potential areas for intervention to facilitate hospitalised patients’ engagement in SDM with healthcare professionals on their antibiotic therapies.

## 2 Materials and methods

### 2.1 Study design and study population

Semi-structured interviews were conducted with adult patients, aged 21 years and above, admitted in the general wards of three largest tertiary hospitals in Singapore, between April 2019 and October 2020. Patients receiving antibiotics and were aware that they were given antibiotics during their hospital stay were included in this study. To ensure maximum variation, patients were purposively recruited from the medical and surgical disciplines, with a good representation from different hospitals. For planning purposes, a sample size of 12–15 interviews per stratum (i.e., medical versus surgical disciplines) was estimated in order to achieve data saturation (24). The conduct and reporting of this study followed the Consolidated Criteria for Reporting Qualitative Research (COREQ) guidelines (25) and this study was approved by the National Healthcare Group Domain Specific Review Board.

### 2.2 Semi-structured interviews

A semi-structured interview guide (Supplementary material 1) was developed by HG (Female, PhD, Epidemiologist) and the questions were designed to explore hospitalised patients’ knowledge, attitudes, perceptions and experiences related to antibiotics and AMR, and their interactions with their clinical team during the hospital stay. Pilot interviews were conducted by a study team member (Female, BSc, Research Assistant) with two staff members from the hospital who had at least one prior hospitalisation experience in a local tertiary hospital to ensure content validity and proper phrasing of the questions. These staff were from the same institution but they were not affiliated with the study team (they were neither from the same department as the study team members nor had working relationships with them). Interviews were predominantly conducted in English but options were available for the conduct of interviews in Mandarin or Malay languages. Three study team members (Females, 2 BSc and 1 MPH, Research Assistants respectively), who were bilingual and trained in qualitative research, conducted the interviews.

Patients were invited in-person by the study team in each hospital after checking with their respective clinical teams on their capacity to be interviewed. Only those who were deemed fit and without cognitive impairment were recruited into the study. Informed consent was taken from interested patients on the same day of the interview and the duration of each interview was around 60 min. The interview was conducted physically at the bedside or virtually via Zoom during the coronavirus disease 2019 (COVID-19) pandemic, with curtains drawn to maintain the confidentiality of the patients. For virtual interviews, the study team would provide the patients with a digital tablet and set up the Zoom session before leaving the bedside to commence the interview. This was to minimise physical contact time with the patients to reduce COVID-19 transmission risks. No personal identifier was collected during the interview and each patient was provided with a unique participant number to be used through the study. Prior to the start of the interview, the interviewer would introduce herself as a public health researcher who did not work under the same department as the clinical team providing hospital care to the patient. This was to assure the patients that the care they received would not be compromised by taking part in the interview, and also to minimise social desirability bias to enhance the truthfulness of the responses received. All interviews were audio-recorded and transcribed verbatim.

### 2.3 Data analysis

Applied thematic analysis was performed (26). HG read and re-read the transcripts to familiarise with the data before developing the codebook according to the TDF (22). The preliminary codebook was reviewed by AC (Female, PhD, Associate Professor) and the final codebook was applied to all transcripts. The domains of TDF were later mapped onto and grouped under the elements of the COM-B model (23) as proposed by Fahim et al. (27). The six broad thematic codes were capability (psychological), capability (physical), opportunity (social), opportunity (physical), motivation (automatic) and motivation (reflective). Any TDF domain not mapped onto the COM-B model was reported as well. Any discrepancies that arose during data analysis were resolved between AC and HG. Data saturation was achieved, in agreement with AC, at around two-thirds way through the interviews. The emergent themes (***bold italics***) and subthemes (*italics*) were organised using Atlas.ti, whilst basic descriptive data were tabulated using Microsoft Excel.

## 3 Results

### 3.1 Study participants

A total of 23 in-depth interviews were conducted (Table 1), with an almost equal representation from medical (43%) and surgical (57%) disciplines. The median age of the participants was 42 (range: 22─69) years, with a slight preponderance of males (61%) and Chinese (57%). Slightly more than half of the participants were lower educated (52%).

**Table 1:** Basic characteristics of the study participants.

Characteristics	Hospital A (*N* = 9)	Hospital B (*N* = 10)	Hospital C (*N* = 4)	Total (*N* = 23)
*Discipline, N (%)*				
Medical	4 (44)	3 (30)	3 (75)	10 (43)
Surgical	5 (56)	7 (70)	1 (25)	13 (57)
*Median age, years (min, max)*	42 (22, 69)	40.5 (25, 63)	48 (42, 55)	42 (22, 69)
*Gender, N (%)*				
Male	4 (44)	6 (60)	4 (100)	14 (61)
*Ethnic group, N (%)*				
Chinese	6 (67)	6 (60)	1 (25)	13 (57)
Malay	1 (11)	3 (30)	1 (25)	5 (22)
Indian	2 (22)	1 (10)	2 (50)	5 (22)
*Residency status, N (%)*				
Singapore citizen	9 (100)	7 (70)	2 (50)	18 (78)
Singapore permanent resident	0	2 (20)	1 (25)	3 (13)
Foreigner	0	1 (10)	1 (25)	2 (9)
*Educational level, N (%)*				
Lower educated (A-levels and below)	3 (33)	7 (70)	2 (50)	12 (52)
Higher educated (Diploma and above)	6 (67)	3 (30)	2 (50)	11 (48)

### 3.2 Qualitative findings

***Capable of actively querying the clinical team on their antibiotic therapies if motivated by perceived negative consequences of antibiotics.***

A small proportion of participants revealed that they would actively query the clinical team on any antibiotic therapy prescribed to them. It was observed that these hospitalised patients were *motivated to raise questions due to side effects of antibiotics experienced in the past, or due to perceived negative outcomes from the use of antibiotics*, such as fear of driving antibiotic resistance.

*“Every time when antibiotics need to be eaten, I feel like rejecting…Because I feel like if I take too much antibiotics, I do not know if I will cause my own immune system to be bad…I complained to the doctor today that my stomach always feels uncomfortable after I eat [antibiotics]…I did ask the doctor why I need to take antibiotics.”* (IDI01, 59yo, Female, Lower educated).

*“I remember a few years back, I had rashes. I was allergic to one of the antibiotics. So I will ask the doctor first. What is the purpose of it, and also if it is going to reoccur? Whatever happened in the past, you know?”* (IDI15, 46yo, Female, Lower educated).

***Not actively querying the clinical team on their antibiotic therapies if lack the motivation and opportunities to do so.***

On the other hand, several reasons were uncovered as to why the majority of the participants did not engage in active conversations with their clinical team on the antibiotic therapy they received. A *self-perceived paternalistic relationship with their clinical team could have diminished these hospitalised patients’ motivation in questioning* the antibiotic therapy received. Additionally, some patients also expressed that they were *less likely to query if they trusted the doctors’ professional know-how and mission to provide the best treatment* for them.

*“For me, if they [referring to the clinical team] give me antibiotics, then I guess I should just take it.”* (IDI06, 28yo, Male, Higher educated).

*“I will eat what the doctor asks me to eat. It is only right to listen to the doctor’s instructions. It is not as if the doctor will want to harm us, right?”* (IDI08, 63yo, Male, Lower educated).

*“From my point of view, I see it as the doctor has the final say. They know what is best for me. I should not need to question them whether this is effective for me or not. Whatever they prescribe, I will take it as it is going to help me. So I will not question further.”* (IDI23, 28yo, Male, Higher educated).

A *self-perception of own knowledge of antibiotics (both good and poor knowledge) also seemed to demotivate* these hospitalised patients in engaging in active conversations with their doctors. Patients, who felt that they had an inadequate knowledge of antibiotics and those who perceived that they had a relatively good knowledge about antibiotics (although not necessarily correct), had shared that they did not raise questions to their clinical teams about their antibiotic therapies.

*“I just do not ask. Because I feel that the clinical team would know better than me…I feel like that they have more knowledge so I just follow their instructions.”* (IDI06, 28yo, Male, Higher educated).

*“We do not go into the details actually. I know generally antibiotics are given to create an immune system in the body for the condition. So I normally do not ask any questions.”* (IDI05, 51yo, Male, Higher educated).

Hospitalised patients also opined that they were *more likely to go with the suggested treatment plan decided by the clinical team due to their desire to recover quickly* from their conditions. This was further *compounded by an absence of bad past experiences with antibiotics* and *a perception of good outcomes from the use of antibiotics*, as highlighted by some patients. As a result, these participants were reportedly less likely to query the clinical team on the need for antibiotics.

*“Because my skin has to take a longer time to heal. Hence, there is no choice but to use antibiotics.”* (IDI03, 55yo, Male, Lower educated).

*“I can feel that my wound is healing faster. My wound dries faster. I like to keep getting antibiotics because I can heal faster.”* (IDI14, 27yo, Male, Lower educated).

*“I am kind of ok with taking it [referring to antibiotics]. I have not had any bad experiences with antibiotics yet.”* (IDI17, 22yo, Male, Higher educated).

*“If the antibiotic helps my body to get better, then I will just take it.”* (IDI19, 31yo, Female, Higher educated).

The *perceived lack of capability to communicate effectively and absence of opportunities for interaction* were potential barriers to hospitalised patients’ engagement in antibiotic conversations with their doctors. Besides language barriers, a couple of participants shared that they would refrain from asking their doctors any questions due to their perceived lack of knowledge and the ability to properly articulate medical-related questions and to adequately understand the explanations given by their doctors. In addition, as observed and reported by participants, doctors would usually be in the wards for short periods of time and during busy ward rounds, limiting the opportunities for patients to interact with their doctors. On the contrary, some participants voiced that there were *more instances to interact with nurses as the latter would usually be around in the ward caring* for them. Notably, a few patients expressed concerns about burdening the medical team with their antibiotic questions, while observing the team’s busy work schedules.

*“I am afraid that I will not understand even after he tells [me about antibiotics]. Because that doctor is a Caucasian. But he knows how to speak some Mandarin…I do not know how to ask him those medical sort of questions.”* (IDI01, 59yo, Female, Lower educated).

*“For questions on what medications to provide, it is always the staff nurses who will discuss with us actually…Maybe the doctors are busy…they spend not even one minute with the patients when they are around.”* (IDI05, 51yo, Male, Higher educated).

Despite so, some participants highlighted that they *desired more opportunities to discuss with their doctors and receive information from them on their antibiotic therapies*. They valued such interactions, as those could help them to understand the rationale for the antibiotics and learn how to use antibiotics properly.

*“I think the doctor should be the one that tells you whether you have a bacterial or viral infection, and then explain the reasons why he will not be giving antibiotics…If it is bacterial infection, then he should explain like, ‘I am giving you this antibiotic because it is a bacterial infection.’ And also explain why we should complete the full course.”* (IDI06, 28yo, Male, Higher educated).

*“It will be best if he [referring to the doctor] can articulate it clearly to us, the patients. To explain the reasons to why an antibiotic is taken and how it helps with the wound. That will help us to understand better. We will not comprehend if the prescription was given without any explanation since it will be rare for us to ask the doctor too.”* (IDI08, 63yo, Male, Lower educated).

*“Every time the doctor is to prescribe the antibiotics, if the nurse or the pharmacist is able to give a verbal explanation…I think that will help. I think that is the most effective way, because when you receive the medicine, obviously we need to know when we need it, how are we supposed to take it, and what are the precautions to take.”* (IDI23, 28yo, Male, Higher educated).

***Limited knowledge of the antibiotic therapy prescribed.***

Interviewed hospitalised patients seemed to have poor knowledge pertaining to the antibiotic therapy prescribed to them. Most of the participants highlighted that they were *generally informed of their prescribed antibiotic therapy through nurses* as it was a common practice to do so, prior to administration of any drug to patients. Through observing what the nurses did, almost all were *aware of the administration route of the antibiotics prescribed* to them and *the dosing frequency of the antibiotic therapy*.

*“When it’s time for medication, the nurses will come in, and they will tell me to take the medication. They generally do not describe what the antibiotics is for, although they do say that it is an antibiotic. They will read out the name too.”* (IDI23, 28yo, Male, Higher educated).

*“Initially they [referring to the prescribed antibiotic] were given* via *drips. Then subsequently the clinical team changed it into tablets.”* (IDI19, 31yo, Female, Higher educated).

*“Every 8 h, the nurse will come again and give me the antibiotic. Every day.”* (IDI18, 42yo, Male, Lower educated).

However, these patients could not share further information on the other details of their antibiotic therapy. While most knew that the prescribed antibiotics were used to treat their bacterial infections, the rest *could not articulate the reasons why antibiotics were prescribed*.

*“Because of the wound, I was given antibiotics. If not, it will get infected.”* (IDI14, 27yo, Male, Lower educated).

*“I think the antibiotics are for my…I do not know. For my inside? And the nebuliser is for my breathing.”* (IDI04, 45yo, Male, Lower educated).

*“I was prescribed antibiotics maybe to protect my gastrointestinal system.”* (IDI10, 69yo, Female, Lower educated).

Furthermore, many interviewed patients were *unaware of the duration of the antibiotic therapy* they were on. It was notable that the majority *could not name the antibiotics they were prescribed* with as well. These patients emphasised that they were either not given the information or they could not remember the names provided.

*“I am not sure how long I need to be on the antibiotic, but I am guessing all the way till next week.”* (IDI17, 22yo, Male, Higher educated).

*“I am not sure what kind of antibiotics are those. I just know it is an antibiotic…I do not know the name of it.”* (IDI19, 31yo, Female, Higher educated).

*“They did tell me the name, but I do not study medicine. I do not remember them.”* (IDI23, 28yo, Male, Higher educated).

Last but not least, only one patient mentioned being *aware of the potential side effects of antibiotics* when consumed.

*“The doctor told me about the side effects for the antibiotic. My doctor said that it is normal to…visit the toilet maybe about 3 or 4 times.”* (IDI07, 25yo, Male, Lower educated).

## 4 Discussion

**Figure 1:**
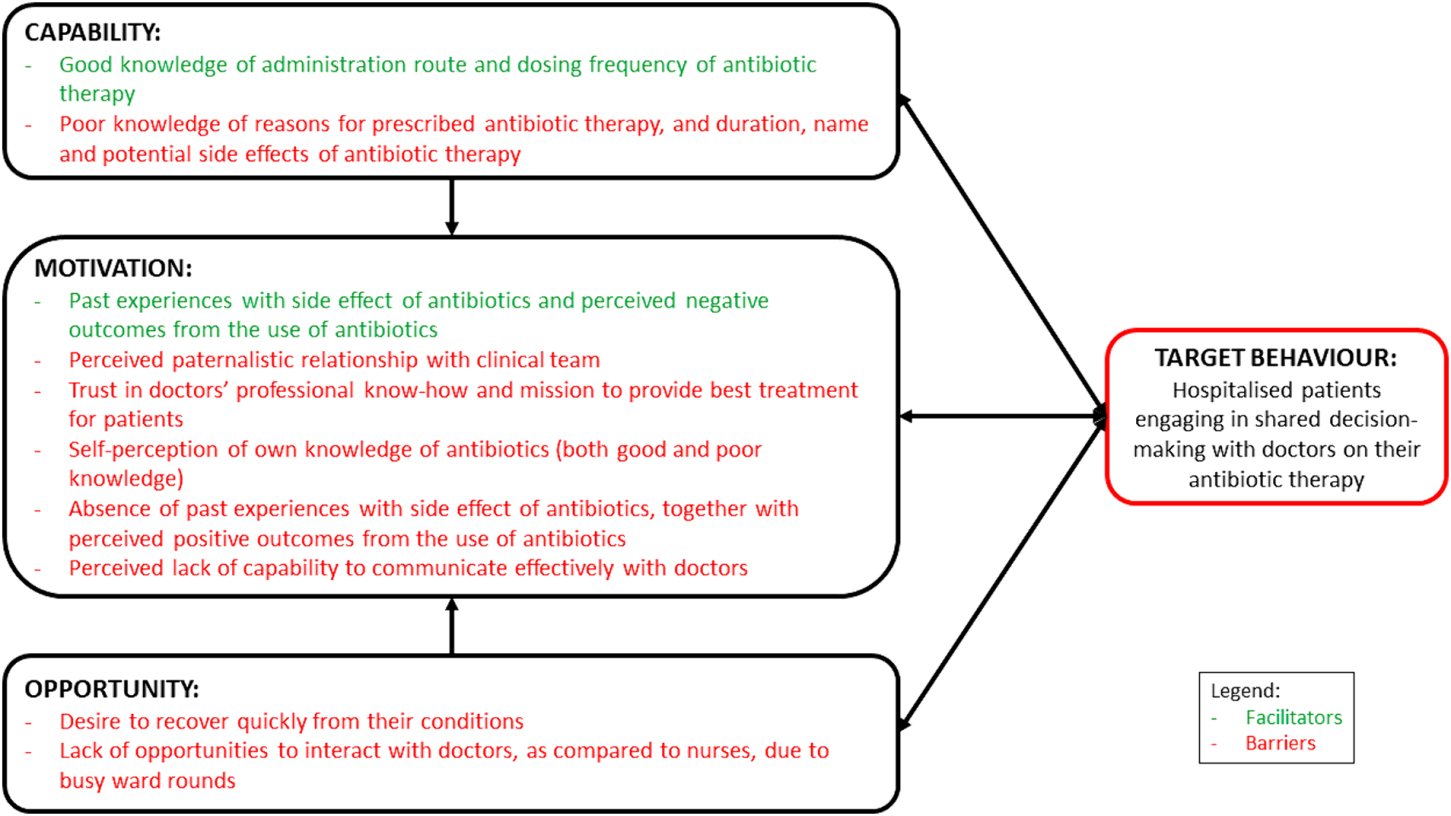
Schematic representation of the facilitators and barriers, mapped on the COM-B model, influencing hospitalised patients to engage in shared decision-making with healthcare professionals on antibiotic use.

This study has provided valuable insights on the facilitators and barriers influencing hospitalised patients’ engagement in SDM with healthcare professionals on antibiotic use in an Asian context (Figure 1). Overall, we observed that patients admitted to tertiary hospitals in Singapore were generally apprehensive about being actively involved in SDM to discuss their prescribed antibiotic therapy with their doctors. This was similarly reported by Zanichelli et al. (21) where they found that a perceived lack of medical knowledge and a trust in doctors’ professional know-how to provide the best treatment plan could impede SDM. In our study, the patients also shared that they were reluctant to initiate conversations with their doctors due to language barriers, and perceived lack of knowledge and the ability to engage in technical conversations surrounding medical science and terminology, which could have resulted in their increased tendency to trust their doctors. Together with the lack of opportunities to meet and communicate with their doctors, unanimously highlighted by our participants, and corroborated with previous literature (20, 21), it is therefore crucial to first tackle communication barriers faced by hospitalised patients. In order to enable and empower hospitalised patients to be more engaged in SDM with their doctors for their prescribed antibiotic therapy, a multi-pronged intervention would be needed.

Leveraging on the facilitators, barriers and desired states identified through the interviews, intervention strategies were recommended using the behaviour change wheel (BCW) (23) – an extension from the COM-B system – to encourage SDM between hospitalised patients and doctors for their antibiotic therapies (Table 2).

**Table 2:** Recommended strategies to engage hospitalised patients in SDM for their prescribed antibiotic therapy.

Desired state by hospitalised patients	Facilitators and barriers influencing engagement in SDM	Recommended strategies to improve patients’ engagement in SDM
Fast recovery and relief of discomfort	Past experiences with side effects of antibiotics Perceived outcomes from the use of antibiotics	***Intervention function: Education and persuasion*** To design patient educational materials to highlight the potential side effects of antibiotic use with specific emphasis on: The potential extension of recovery period due to the additional side effects of antibiotic use; and The undesirable physical discomforts that would accompany with the side effects
More opportunities to discuss and receive information from doctors	Paternalistic relationship with clinical team Trust in doctors’ professional know-how and mission to provide best treatment Afraid of being burdensome by querying clinical team	***Intervention function: Enablement, persuasion and environmental restructuring*** To encourage open communication between the clinical team and patients by: Introducing a change in work culture for clinical team to expect and receive queries from patients when they are in doubt of their antibiotic therapy; Having the clinical team to encourage patients to ask questions when they have doubts on their antibiotic therapy; Designing and creating visual cues to assure and nudge patients to actively approach the clinical team to clarify any doubts pertaining to their antibiotic therapy; and Measuring patient satisfaction and related indicators to encourage active discussions between the clinical team and patients around their antibiotic therapy
	Lack of capability and opportunities to communicate effectively with doctors More opportunities to interact with nurses	***Intervention function: Environmental restructuring*** To expand nurses’ roles to become antibiotic stewards and upskill them to: Receive and relay queries on antibiotic use from patients to their attending doctors; Provide accurate information to the patients for basic questions related to antibiotic administration; and Ensure patients’ queries are answered during hospital rounds by the doctors
Good knowledge of antibiotic use	Good knowledge of administration route and dosing frequency of prescribed antibiotics Poor knowledge of reasons for use, duration, name and associated side effects of prescribed antibiotics	***Intervention function: Education*** To provide patients with bedside patient information leaflets delivered through offline or online modalities on the details of the antibiotic therapy prescribed, which include: Name of antibiotic prescribed; Route of administration; Duration and dosing frequency; Reason(s) for prescribing the antibiotic; and The side effect(s) of the antibiotic prescribed

First and foremost, it was noted that side effects or allergic reactions experienced with antibiotics in the past had played an important role in motivating hospitalised patients to engage in SDM. The negative experiences, together with perceived negative outcomes from antibiotic use, had prompted these patients to proactively ask their doctors about their antibiotic therapies. However, it is the opposite phenomenon ─ patients who had never experienced or never knew of the side effects of antibiotics and therefore were less likely to ask ─ that warrants attention. The techniques of persuasion can be used to develop relevant educational materials to highlight the potential side effects of antibiotics. It was suggested by a systematic review that such a multifaceted approach could provide better outcomes in behaviour change than providing education alone (28). Messages should relate to their desired state of fast recovery and relief of discomfort and how potential side effects of antibiotics could jeopardise that. This could thereafter spark interest amongst hospitalised patients to want to understand more about the antibiotics prescribed to them and their related side effects from their doctors.

Next, despite patients’ desire for more opportunities to discuss and receive antibiotic-related information from their doctors (20, 21), a paradigm shift would be required to encourage open communications between patients and their clinical teams. For a start, the hospital environment needs to be restructured to create an enabling working culture for the clinical team to be receptive to receiving questions from their patients. Coupled with the receipt of patient educational materials, patients could be encouraged by the clinical team to raise any questions when they have doubts pertaining to their prescribed antibiotic therapy. Following the findings from a hand hygiene campaign organised in an acute care hospital in United Kingdom (29), empowering hospitalised patients to voice their concerns to healthcare professionals could result in positive effects on appropriate antibiotic prescribing. To reinforce these cultural changes, the creation of visual cues, such as a badge worn by members on the clinical team (for example, nurses), can be designed while serving two purposes. Firstly, by embedding ‘Ask Me About Your Antibiotics’ on the badge, it could help to nudge and create a channel for hospitalised patients to approach and pose any antibiotic-related questions to any clinical team member who is wearing the badge. Secondly, by adding a secondary declaration that ‘I Am An Antibiotic Steward’ on the same badge, it could remind and motivate clinical team members to be committed towards having active discussions with patients around antibiotic therapies (30). However, even though patients desire a two-way conversation with their doctors (31), the paternalistic model of care is deeply ingrained and adopted by doctors in the Southeast Asian cultural context (32). Furthermore, patients often express their respect and trust for their attending doctors by complying with their recommendations without openly questioning their treatment decisions. As a result, such paradigm shift could be difficult to implement from a ground-up approach. To effect change at the clinical care team level, engagement of the hospital’s management to provide top-down guidance and strategic directions on patient activation would be necessary.

In particular, nurses, by nature of their job, have a high frequency of interaction with hospitalised patients and they are valuable members of the clinical team that should be counted on to improve antibiotic stewardship in tertiary hospitals. The expansion of nursing roles was first introduced by the US Centers for Disease Control and Prevention (33) and broadly discussed by several international studies (34). Involving nurses as champions to don the badge to advocate for patient empowerment in SDM engagement would therefore be ideal. Even though nurses are not empowered to provide clinical advice pertaining to antibiotic choices (35), they are exemplary antibiotic administrators who are well aware of the name, and correct antibiotic dose and duration to be given to the patients for patient safety reasons (36, 37). Once doctors have selected the antibiotics for treatment, nurses could provide patients with information on the indication, choice, dosing frequency, duration and route of administration of the antibiotics. Where further clarifications are required, nurses could direct the patients’ queries to the attending doctors and remind doctors to provide their responses during the next consultation round. Already, nurses are acting as information brokers who actively relay patient’s clinical status to doctors (38). Hence, as supported by previous findings, nurses can potentially take on an expanded role in antibiotic stewardship if they were provided with a formal recognition, proper education and clear guidelines (38, 39).

Last but not least, as recommended by Elwyn et al. (40) and supported by Waddell et al. (41), it is essential to provide hospitalised patients with high quality information before engaging them in SDM. The timely provision of patient information leaflets, through both offline and online modalities, upon prescribing the antibiotic therapy at the bedside would be useful to increase their awareness and knowledge of the antibiotics administered. The availability of both offline and online resources is important to ensure sufficient reach to the intended audience due to differential acceptance for digital materials by different age groups (42, 43). The sharing of the patient information leaflets should also be accompanied by a brief explanation given by a doctor or a trained nurse on the key messages, as provider-facilitated education is likely to be more effective than self-directed learning (44–46). Information pertaining to the antibiotic therapy can include the name of the antibiotic, route of administration, duration of therapy, dosing frequency, reasons for prescribing, and potential side effects, as recommended by the US CDC in its 2019 updated guidance document (47). This information can also help hospitalised patients to be more involved in their prescribed antibiotic therapy by being mindful of the specific antibiotics administered, and be watchful of the potential side effects expected and highlight them to the clinical team when needed. Nonetheless, the fidelity of this strategy depends on the clinical and cognitive statuses of the hospitalised patients to be able to receive and process the educational messages and visual nudges (i.e., the badge).

There are several strengths to this study. To our knowledge, this is the first qualitative study conducted with hospitalised patients to explore the facilitators and barriers influencing their engagement in SDM on antibiotic therapy. The purposive sampling of patients from both medical and surgical wards had ensured a good representation of voices from the two predominant disciplines within the tertiary hospital setting, where antibiotic prescribing behaviours can be very different. Social desirability bias was highly unlikely as the participants were assured that their participation would not influence the standard of care they received. However, there was a smaller proportion of participants recruited from Hospital C. This was due to unforeseen circumstances that prevented the study team from crossing between institutions and entering a different hospital during the COVID-19 pandemic. Nonetheless, data saturation was achieved despite this shortcoming. Lastly, there is a paucity of evidence-based effective strategies for engaging hospitalised patients in antibiotic stewardship (48). Further efficacy and effectiveness studies should be conducted to assess the feasibility of the proposed strategies. Also, since the recommended strategies are based on the study’s findings in our hospital, further research is warranted to understand the contextual factors in other institutions, for the successful implementation of these interventions.

## 5 Conclusion

Hospitalised patients could be part of the antibiotic stewardship efforts in tertiary hospitals. Multi-pronged strategies focusing on creating an enabling hospital environment and culture, expanding nurses’ role in antibiotic stewardship and dissemination of antibiotic-related educational messages valued by patients, could help to empower patients to be involved in SDM surrounding their prescribed antibiotic therapy.

## Data availability statement

The raw data supporting the conclusions of this article will be made available by the authors, without undue reservation.

## Ethics statement

The studies involving humans were approved by the National Healthcare Group Domain Specific Review Board, Singapore. The studies were conducted in accordance with the local legislation and institutional requirements. The participants provided their written informed consent to participate in this study.

## Author contributions

HG: Formal analysis, Methodology, Project administration, Writing – original draft. DCL: Writing – review & editing. TMN: Writing – review & editing. JS: Writing – review & editing. ALHK: Writing – review & editing. SJC: Writing – review & editing. AC: Conceptualization, Funding acquisition, Methodology, Supervision, Writing – review & editing.
