# Supplementary material for: Hospitalised patients as stewards of their own antibiotic therapy: a qualitative analysis informing the strategic design of interventions to encourage shared decision-making in tertiary hospital settings in Singapore
Source: Front Public Health. 2024 Jul 31;12:1347764. doi: 10.3389/fpubh.2024.1347764 (PMC11322571; doi:10.3389/fpubh.2024.1347764)
Supplement: Supplementary file 1 [file Data_Sheet_1.docx]

**In-Depth Interview Guide**

- What is the reason for your stay in this hospital?
- Would you mind sharing some details of staying in the ward for this hospital stay?
  - How did you relay your condition to the doctor or nurse?
  - What did he/she advise you?
  - How did you feel when he/she provided you with his/her medical advice?
  - Were you aware of what medications were prescribed to you? Would you mind naming them?
- What do you know about antibiotics?
- Were you aware that you were prescribed with antibiotics?
  - Do you know what is the name of the antibiotic(s) given, and how often and how long you would need to be on the antibiotic(s)?
  - How would you describe your thoughts when the doctor mentioned that he/she will prescribe antibiotics to you?
  - Did you mention anything to him/her after hearing that? Why?
  - Did you discuss the need for antibiotics with the doctors or nurses? Why?
  - What’s your impression of the antibiotics given to you? Why?
- Would you mind sharing if this is your first time using antibiotics or you have used antibiotics before?
- [If participant had used antibiotics before] Would you mind sharing your past experience(s) using antibiotics?
  - Why were you prescribed with antibiotics?
  - How did you feel symptomatically after taking the antibiotics?
  - How has/have your previous experience(s) of using antibiotics affected your impression and use of antibiotics now?
